# Is the use of vaginal progesterone a factor in pregnant women diagnosed with gestational diabetes mellitus?

**DOI:** 10.1590/1806-9282.20250444

**Published:** 2025-09-19

**Authors:** Ayşe Rabia Kanbak, Deniz Can Öztekin

**Affiliations:** 1 Bakırçay University, Gynecology and Obstetrics Clinic, Faculty of Medicine – İzmir, Turkey.

**Keywords:** Gestational diabetes, Premature birth, Progesterone

## Abstract

**OBJECTIVE:**

The aim of this study was to investigate the effect of vaginal progesterone used to prevent spontaneous preterm birth in the development of gestational diabetes mellitus.

**METHODS:**

This is a cross-sectional study that investigated pregnant women aged 18–39 years. The study participants underwent a 2-h, 75-g oral glucose tolerance test at 24 and 28 weeks of gestation. A total of 3,066 patients met the inclusion criteria, and 418 were diagnosed with gestational diabetes mellitus based on at least one abnormal plasma glucose value during screening. The remaining 2,648 patients, who had normal plasma glucose levels, were classified as the control group. The two groups were compared based on various factors, including age, parity, pre-pregnancy body mass index, smoking status, gestational age, and use of vaginal progesterone.

**RESULTS:**

The use of vaginal progesterone was statistically significant at a higher rate in the gestational diabetes mellitus group compared to the control group (22.0 vs. 16.0%; p=0.002). The mean duration of vaginal progesterone use was 53.4±14.6 days (ranging from 28 to 90 days), and this duration was statistically significantly longer in the gestational diabetes mellitus group (59.9±14.8 vs. 52.0±14.2; p<0.001).

**CONCLUSION:**

The findings suggest that the use of vaginal progesterone may increase the risk of gestational diabetes mellitus, and the risk appears to rise with the duration of progesterone use. It is important to consider that patients on prolonged vaginal progesterone, particularly those started early in pregnancy, may be at increased risk of diabetes. Therefore, it may be advisable to repeat the oral glucose tolerance test performed at 24–28 weeks in the following weeks.

## INTRODUCTION

Gestational diabetes mellitus (GDM) is an important metabolic disorder during pregnancy that affects maternal and fetal health, both immediately and in the long term. It affects approximately 17% of the pregnant population worldwide^1,2^.

During pregnancy, physiological changes such as fasting hypoglycemia, postprandial hyperglycemia, hyperinsulinemia, and heightened insulin resistance occur^3^. Hormones such as cortisol and estrogen exacerbate insulin resistance, which becomes more pronounced as pregnancy progresses^4^. As the placenta grows and secretes increasing amounts of hormones, the pancreas may fail to produce sufficient insulin to maintain normal glucose levels, resulting in GDM. Women with normal glucose tolerance prior to pregnancy who develop GDM may have underlying glucose metabolism irregularities^5^.

Preterm birth (PTB) is birth before the 37th week of pregnancy. It increases neonatal mortality and morbidity and accounts for approximately 10% of all births^6^. The priority in the clinical approach is to prevent PTB^6^. Women who have previously given birth prematurely have an increased risk of having a similar preterm birth at a similar gestational age in subsequent pregnancies^7^. These pregnancies have a five- to six-fold increased risk compared to the normal population without a history of PTB^8^. In addition, a cervical length shorter than 25 mm measured before 24 weeks is a predictor of preterm birth^9^.

In women with a previous history of SPTB, vaginal progesterone administration has been shown to prevent SPTB in singleton pregnancies, whether or not cervical shortness is present^10^. Vaginal progesterone use is recommended for pregnancies meeting these criteria^11^.

While 17-alpha-hydroxyprogesterone caproate (17OHP-C), a form of progesterone used to prevent SPTB, increased the risk of GDM, and the diabetogenic impact of vaginal progesterone remains unclear despite its growing popularity in preventing SPTB^12–14^.

The purpose of this study was to investigate the relationship between vaginal progesterone use for SPTB prevention and the development of GDM.

## METHODS

This cross-sectional study included pregnant women aged 18–39 years who visited our clinic between September 1, 2018, and January 1, 2023, and underwent a 24–28 week, 75-g OGTT.

Patient data were screened from hospital databases. Of the 3,981 pregnant women screened, pre-pregnancy and early gestational age data were not available for 241. Additionally, 457 pregnancies were excluded due to pre-gestational diabetes, previous diagnosis of GDM, delivery of a baby weighing more than 4,000 g, pre-pregnancy body mass index (BMI) ≥30, or conception using assisted reproductive technology (ART). Notably, 217 women were excluded if they used vaginal or other forms of progesterone for less than 4 weeks.

After patients were eliminated according to the exclusion criteria, a total of 3,066 patients were included in the study. In total, 418 patients were diagnosed with GDM due to at least one high plasma glucose value in 75 g OGTT. The control group consisted of 2,648 patients with normal glucose levels. These two groups were compared. The parameters calculated include age, parity, BMI before pregnancy, smoking status, gestational week, and vaginal progesterone use.

**Figure 1:**
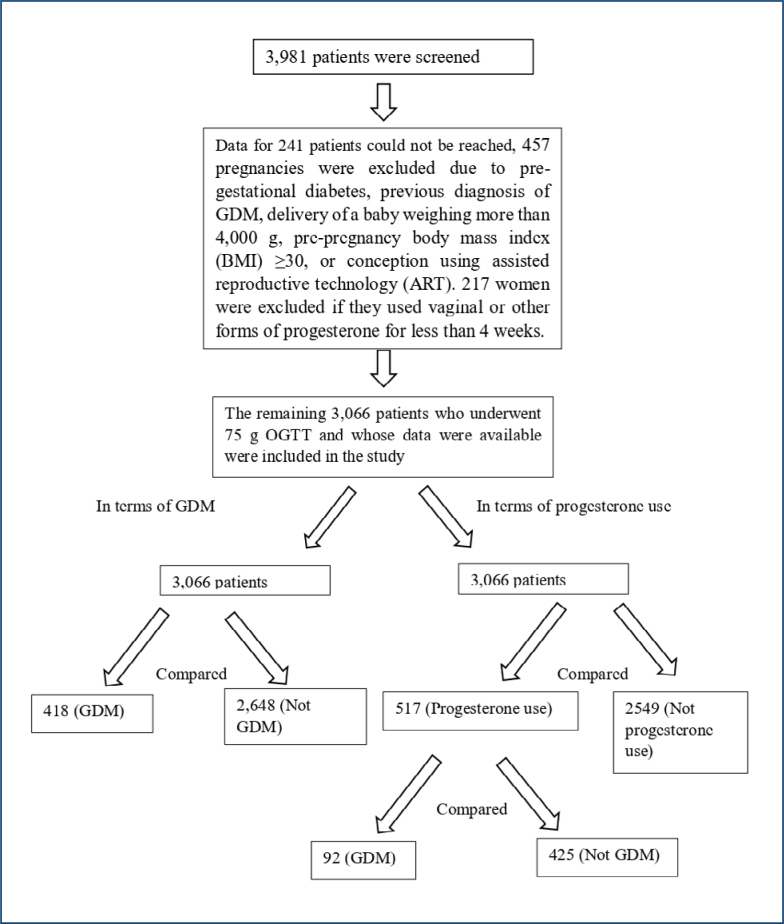
Study flow chart.

Additionally, 3,066 patients were evaluated in two groups as those using and those not using progesterone. The vaginal progesterone group (200 mg micronized progesterone per day) for at least 4 weeks was categorized as the "progesterone treatment" group, while others were designated as "never treated." OGTT results were compared between these groups. Notably, 517 patients using progesterone and 2,549 patients not using progesterone were compared in terms of GDM. A subgroup analysis was performed for the 517 patients using progesterone, including 92 patients diagnosed with GDM and 425 patients not diagnosed with GDM in terms of indications for progesterone use, timing of treatment initiation, duration of treatment, and cervical length (Figure 1).

GDM was diagnosed with the International Association of Diabetes and Pregnancy Study Groups (IADPSG) criteria. These are fasting plasma glucose (FG) ≥92 mg/dL, 1-h plasma glucose (PG) ≥180 mg/dL, or 2-h PG ≥153 mg/dL. A high level of only one of these three values diagnoses GDM^15–17^.

Before OGTT, no patient received intramuscular corticosteroids for fetal lung maturity.

Gestational week was calculated from the last menstrual period and confirmed by fetal crown-rump length during the first trimester ultrasound.

Cervical shortness (≤25 mm) was determined by combining vaginal ultrasound and fetal anatomic scanning between 18 and 22 weeks^18^.

Statistical analysis results are presented as mean ±SD for continuous variables and numbers (percentages) for categorical variables. Data were analyzed using the χ^2^ test and student's t-test. A p-value <0.05 was considered statistically significant.

Assuming a general GDM prevalence of approximately 17%, our sample size provided 80% power to detect a 50% increase in GDM incidence in the progesterone-treated group at a 1:4 case-to-control ratio. Post-study power analysis with α=0.05 indicated 83% power.

The ethics committee approved this study (approval number 854, January 11, 2023).

## RESULTS

The overall GDM incidence was 13.6%, potentially underreported due to the exclusion of high-risk patients. Demographic data (Table 1) revealed no significant differences in mean age between groups (29.1±6.6 vs. 29.4±6.1; p=0.35). However, vaginal progesterone usage was significantly higher in the GDM group compared to controls (22.0 vs. 16.0%; p=0.002).

**Table 1:** Demographic characteristics of gestational diabetes mellitus and control groups.

	GDM (n=418)	Control (n=2,648)	p-value
Age (year)	29.1±6.6	29.4±6.1	0.35
Nulliparity	236 (56.4%)	1,475 (55.7%)	0.77
Pre-pregnancy BMI (kg/m^2^)	28.4±7.4	28.8±8.1	0.34
Smoking	59 (14.1%)	398 (15.0%)	0.62
Gestational age (weeks)	25.5±2.1	25.4±1.3	0.18
Vaginal progesterone	92 (22.0%)	425 (16.0%)	0.002*

Data are presented as mean±standard deviation or numbers (percentage). GDM: gestational diabetes mellitus; BMI: body mass index. p<0.05 was accepted as statistically significant.

* statistically significant.

Subgroup analysis showed that prior PTB was a more common indication for progesterone use in the GDM group (56.5 vs. 46.1%), while cervical shortening was less common (33.7 vs. 44.9%). Progesterone therapy began earlier in the GDM group (mean: 18.6±2.8 weeks vs. 19.1±2.6 weeks; p=0.11) but was not statistically significant. Progesterone usage prior to 20 weeks was more frequent among GDM patients (63.0 vs. 55.2%; p=0.17). Duration of progesterone use was longer in the GDM group (59.9±14.8 days vs. 52.0±14.2 days; p<0.001) (Table 2).

**Table 2:** Parameters related to progesterone usage in gestational diabetes mellitus and control group.

	GDM (n=92)	Control (n=425)	p-value
Age (year)	29.8±9.4	29.6±7.6	0.82
Vaginal progesterone indications			0.14
Short cervix	31 (33.7%)	190 (44.9%)	
History of SPTB	52 (56.5%)	196 (46.1%)	
Both	9 (9.8%)	39 (9.0%)	
Initiation of progesterone usage (weeks)	18.6±2.8	19.1±2.6	0.11
Initiation of progesterone <20 weeks	58 (63.0%)	235 (55.2%)	0.17
Duration of progesterone usage (days)	59.9±14.8	52.0±14.2	<0.001*
Cervical length (mm)	25.5±5.8	26.4±7.1	0.25

Data are presented as mean±standard deviation or numbers (percentage). GDM: gestational diabetes mellitus; SPTB: spontaneous preterm birth. p<0.05 was accepted as statistically significant.

* statistically significant.

A significantly higher GDM rate was observed in the progesterone-treated group compared to the untreated group (17.7 vs. 12.7%; p=0.002; OR 1.47, 95%CI 1.14–1.90). Mean OGTT values were higher in progesterone-treated patients, although fasting, 1-h, and 2-h plasma glucose differences were not statistically significant (Table 3).

**Table 3:** Comparison of 2-h, 75-g oral glucose tolerance test results between women with and without vaginal progesterone treatment.

	Progesterone treated (n=517)	None treated (n=2,549)	p-value
Fasting plasma glucose (mg/dL)	88.1±9.4	87.6±9.1	0.26
1-h plasma glucose (mg/dL)	162.6±37.5	161.6±32.5	0.53
2-h plasma glucose (mg/dL)	141.6±32.4	139.4±26.8	0.10
GDM	92 (17.7%)	326 (12.7%)	0.002*

Data are presented as mean±standard deviation or numbers (percentage). GDM: gestational diabetes mellitus. p<0.05 was accepted as statistically significant.

* statistically significant.

## DISCUSSION

Preterm birth (PTB) is linked to higher rates of neonatal mortality and long-term health complications^6^. Numerous studies have compared the effectiveness of oral, intramuscular 17-alpha hydroxyprogesterone caproate (17OHP-C), and vaginal progesterone formulations in preventing PTB^19,20^. Recent research, however, has demonstrated that vaginal progesterone is more effective in preventing PTB^21,22^. Based on these findings, our clinic prefers vaginal progesterone to prevent SPTB, due to its ease of use. Available evidence supports the safety of starting vaginal progesterone therapy after the 14th week of pregnancy for both the mother and fetus^23^.

According to the American College of Obstetricians and Gynecologists (ACOG) guidelines, women with a history of SPTB or those with a short cervix detected on ultrasound between 18 and 22 weeks of pregnancy should begin treatment with vaginal progesterone at 14 weeks of gestation. The therapy continues until the 37th week or delivery^11,24^.

While some studies suggest that progesterone used to prevent PTB may increase the risk of GDM, other studies show conflicting results. A meta-analysis indicates that women using 17OHP-C to prevent PTB have a higher risk of GDM, but the evidence regarding vaginal progesterone remains inconclusive^12^.

The OPTIMUM study evaluated GDM rates as a secondary outcome. Patients who started progesterone treatment at 22–24 weeks were included. The results showed no significant difference in GDM rates between the vaginal progesterone group (4.6%) and the placebo group (6.3%)^23^.

In another study, it was found that weekly 17OHP-C administration was not associated with higher GDM rates in either singleton or twin pregnancies^25^. Concerning vaginal progesterone, a study concluded that daily vaginal progesterone use does not increase the likelihood of abnormal glucose load testing or GDM, and that additional GDM screening or diagnostic tests outside the standard schedule are unnecessary unless other risk factors are present^13^. Similarly, another study found no increased risk of GDM associated with vaginal progesterone use for preventing recurrent preterm births or in women with a short cervix^14^.

In contrast to previous studies, our research found a significant association between vaginal progesterone use and a higher incidence of GDM when comparing PTB prevention methods. Additionally, when comparing progesterone users to non-users, the GDM rate was significantly higher among progesterone users.

The diabetogenic effect of 17OHP-C is attributed to its systemic influence on carbohydrate metabolism following intramuscular administration. This effect is not observed with vaginal progesterone, which acts locally and is believed to have minimal systemic side effects. While systemic effects of vaginal progesterone are thought to be limited, it is not entirely devoid of impact on carbohydrate metabolism. The key factors influencing this issue are the duration of use and the administration route.

In a previous study involving a smaller patient group, we observed that vaginal progesterone use for SPTB prevention during pregnancy was linked to a higher risk of GDM, especially when treatment started early and lasted for a longer duration^26^. In this larger-scale study, we confirmed that vaginal progesterone use for preventing SPTB increases GDM risk. While no direct relationship was found between the timing of vaginal progesterone initiation and GDM risk, a significant correlation was identified between the duration of use and the increased risk of GDM.

The limitations of our study are its retrospective nature and the small number of patients. Randomized controlled trials with larger sample sizes are needed to clarify these findings. Another limitation is that, as a tertiary center, many patients gave birth in different hospitals, making it difficult to follow them until delivery. This adds complexity to the results of our study.

## CONCLUSION

Our findings suggest that prolonged vaginal progesterone use for SPTB prevention is associated with increased GDM risk. Patients initiating therapy earlier in pregnancy may require closer glucose monitoring. We recommend repeating OGTT after 24–28 weeks in prolonged users. Larger, randomized controlled studies are needed to confirm these observations and inform clinical guidelines.

## Data Availability

The datasets generated and/or analyzed during the current study are available from the corresponding author upon reasonable request.
